# Diethylstilbestrol and autism

**DOI:** 10.3389/fendo.2022.1034959

**Published:** 2022-11-21

**Authors:** Marie-Odile Soyer-Gobillard, Laura Gaspari, Philippe Courtet, Charles Sultan

**Affiliations:** ^1^ Univ Sorbonne, Centre National de la Recherche Scientifique (CNRS), Paris, France; ^2^ Association Halte aux HORmones Artificielles pour les GrossessES (Hhorages)-France, Perpignan, France; ^3^ Centre Hospitalier Universitaire (CHU) Montpellier: Univ Montpellier, Unité d’Endocrinologie-Gynécologie Pédiatrique, Service de Pédiatrie, Montpellier, France; ^4^ Centre Hospitalier Universitaire (CHU) Montpellier: Univ Montpellier, Centre de Référence Maladies Rares du Développement Génital, Constitutif Sud, Hôpital Lapeyronie, Montpellier, France; ^5^ Univ Montpellier, Institut National de la Santé et de la Recherche Médicale (Inserm) 1203, Développement Embryonnaire Fertilité Environnement, Montpellier, France; ^6^ Institut de Génomique Fonctionnelle (IGF), Univ. Montpellier, Centre National de la Recherche Scientifique (CNRS), Institut National de la Santé et de la Recherche Médicale (Inserm), Montpellier, France; ^7^ Department of Emergency Psychiatry and Acute Care, Lapeyronie Hospital, Centre Hospitalier Universitaire (CHU) Montpellier, Montpellier, France

**Keywords:** diethylstilbestrol, psychiatric disorders, autism, epigenetics, endocrine disruptors

## Abstract

It is acknowledged that diethylstilbestrol (DES), a synthetic diphenol with powerful estrogenic properties, causes structural anomalies of the reproductive tract and increases the risk of cancer and genital malformations in children and grandchildren of mothers treated during pregnancy. Conversely, data on DES effects on neurodevelopment and psychiatric disorders in *in-utero* exposed children and their descendants are rare, especially concerning Autism Spectrum Disorders (ASD). Recent studies presented in this review strengthen the hypothesis that *in-utero* exposure to DES and also other synthetic estrogens and progestogens, which all are endocrine disruptors, contributes to the pathogenesis of psychiatric disorders, especially ASD. A large epidemiological study in the USA in 2010 reported severe depression in *in-utero* exposed children (n=1,612), and a French cohort study (n=1,002 *in-utero* DES exposed children) in 2016 found mainly bipolar disorders, schizophrenia, major depression, suicide attempts, and suicide. Few publications described ASD in *in-utero* exposed children, mainly a Danish cohort study and a large Chinese epidemiological study. Molecular studies on endocrine disruptors demonstrated the transgenerational induction of diseases and DES epigenetic impact (DNA methylation changes) at two genes implicated in neurodevelopment (*ZFP57* and *ADAM TS9*). We recently described in an informative family, somatic and psychiatric disorders in four generations, particularly ASD in boys of the third and fourth generation. These data show that the principle of precaution must be retained for the protection of future generations: women (pregnant or not) should be extremely vigilant about synthetic hormones.

## Introduction

Diethylstilbestrol (DES), a synthetic diphenol with powerful estrogenic properties, is considered the culprit of many somatic disorders in children and grandchildren of mothers treated during pregnancy (1). It has been estimated that worldwide, ~10 million people have been exposed to DES (2). However, very few studies investigated DES effects on neurodevelopment and psychiatric disorder occurrence, especially Autism Spectrum Disorders (ASD), in exposed children and their descendants. A previous review that included a small number of epidemiological studies reported inconclusive results on the risk of psychiatric disorders in DES-exposed individuals (3). On the other hand, O’Reilly et al. (4) analyzed data from a large US epidemiological study (the Nurses’ Health Study), on 76,240 American women among whom 1,612 were exposed to DES *in utero*. They found that the occurrence of major depressive and anxiety disorders was significantly increased (by a factor of 1.47) in exposed women. Two other studies reported psychiatric effects, particularly schizophrenia, bipolar disorders, depression, eating disorders, suicide attempts, and suicide, in adolescent/adults who were *in-utero* exposed to DES (5, 6) and to progestins (7).

In humans, ASD, a neurodevelopmental disorder, is characterized by impaired social interaction, language and communication and also stereotypical behavior (8). In the last years, several studies have brought insights into ASD genetics (9)and epigenetics (10); however, very few works have investigated the relationship between ASD and *in-utero* exposure to synthetic estrogens and/or progestins. Specifically, Zou et al. (11) demonstrated that in rats, prenatal exposure to levonorgestrel (LNG; a synthetic progestin used in oral contraceptive pills) inhibits estrogen receptor ERβ expression in the amygdala through increased methylation of its promoter. ERβ downregulation led to decreased expression of superoxide dismutase 2 and estrogen-related receptor α, and subsequently triggered damage in amygdala tissue through oxidative stress and dysfunction of mitochondria and fatty acid metabolism. These effects contributed to the autism-like behavior observed in the *in-utero* exposed offspring. This suggests that upon LNG exposure, ERβ downregulation in the amygdala during neurodevelopment may contribute to autism-like behavior. Similarly, Li et al. (12) in a large study on a human population found that prenatal progestin exposure was associated with ASD (see section 1.3). According to Perrotti (13), ERs and progesterone receptors (PRs) function as ligand-dependent transcription factors and their distinctive transcriptional activities are initiated when they bind to their respective ligands, like other members of the nuclear hormone receptor superfamily. The ER and PR signaling pathways are modulated through epigenetic mechanisms.

In recent decades, ASD prevalence has significantly increased. This indicates that besides heritability and genetic background, other factors might be implicated in these disorders, particularly environmental factors that influence the central nervous system (CNS) development. Several toxic compounds have been linked to ASD risk, such as heavy metals, air pollutants, pesticides, polycyclic aromatic hydrocarbons and polybrominated diphenyl ethers. In this review, we present studies suggesting that exposure to DES and other synthetic sexual hormones also might contribute to the increasing ASD prevalence.

## Methods

A systematic review of the literature was carried out by searching the PubMed and Google Scholar databases for the period from 2010 to 2022 following the PRISMA guidelines. The chosen starting year was 2010 because it was the year of publication of the study by Titus-Ernstoff et al. (14) showing for the first time DES multigenerational effect in humans, although it only concerned somatic disorders. Only the study by Colborn published in 2004 (15) was added, as a tribute. Studies were identified using the following keywords: diethylstilbestrol, diethylstilbestrol and ASD, progestins, progestins and ASD, psychosis and endocrine disrupting compounds, estrogens and epigenetics. Publications that were not articles (e.g. conference abstracts) and publications that were not in English were excluded.

### Psychiatric disorders in children *in-utero* exposed to DES

Although DES has not been administered to pregnant women since 1971 (USA) and 1977 (France), it continues to wreak havoc in the subsequent generations. Moreover, it was replaced by ethinylestradiol (EE), which cannot be used during pregnancy since 1980, and by synthetic progestins that are still marketed with EE, particularly in contraceptive pills. Synthetic hormones, such as DES, EE, synthetic progestins, were prescribed often as a cocktail not only to women who had a miscarriage or were at risk of miscarriage, but also to those in discomfort or even as a “morning after pill”.

**Figure 1:**
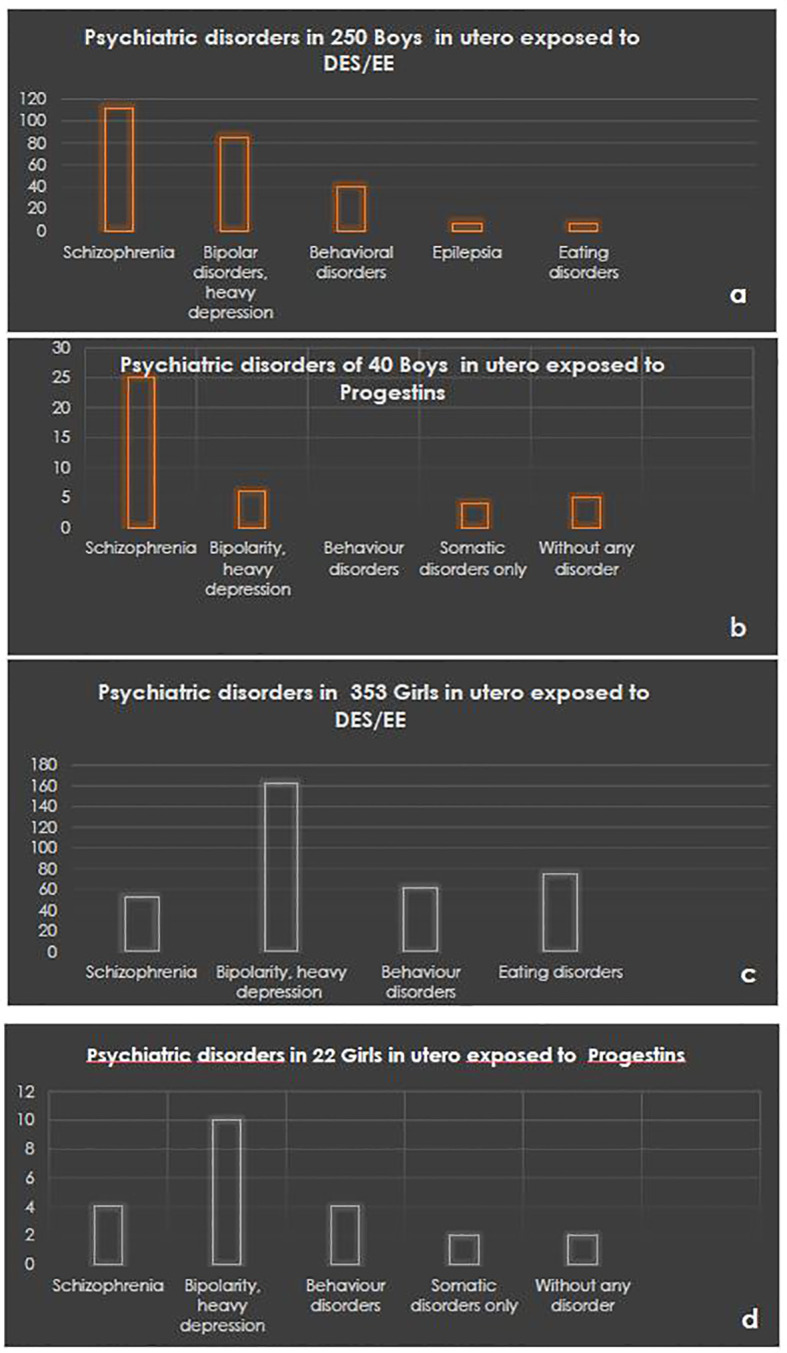
Psychiatric disorder rates in boys *in-utero* exposed to DES/EE **(A)** or to progestins **(B)** and in girls exposed to DES/EE **(C)** or to progestins **(D)**. ^©^Hhorages-France data.

The database of the families of the French patient association Hhorages-France allowed assessing the causal link between *in-utero* exposure to synthetic hormones, particularly DES, and the occurrence of severe psychotic disorders after adolescence in exposed children. This database is based on the responses to a detailed questionnaire written by physicians and researchers and approved by the French national commission for data protection and freedom (CNIL) (7). In 2016, we analyzed data on 1,182 pregnancies from 529 mothers from the Hhorages-France cohort (**Table 1**). We found that 603/720 children exposed to DES *in-utero* and 16 post-DES children (i.e. born after a previous exposed pregnancy but without *in utero* exposure) had psychiatric disorders, but none of the 180 children born before the pregnancy with DES treatment. Compared with the general population, the prevalence of psychiatric disorders in this group was significantly increased (**Table 1b**) (5). Other synthetic hormones, such as EE and progestogens, also are implicated. For instance, *in-utero* exposure to estrogens or progestins increases the risk of bipolar disorders/depression and schizophrenia (**Figure 1**) (16, 17). Patients with bipolar disorder or depression often present also eating disorders (bulimia, anorexia) (**Table 1**). In the Hhorages cohort (5), 30% of children *in-utero* exposed to DES had eating disorders (n =83 vs n=257 with bipolar depression, depression, or anxiety disorder alone). This high rate suggests a possible endocrine cause for this pathology the vulnerability factors of which are still incompletely known (18). Similar observations where reported also in other studies (in the general population) where eating disorders were often associated with major depressive disorders (19, 20). Specifically, Liu et al. (21) carried out a genome-wide association study in which they compared genomic data on 184 patients with bipolar disorder comorbid with eating disorders, 1,370 healthy controls, and 2,006 patients with bipolar disorder only. They found a genome-wide association of eating disorders and bipolar disorders and confirmed the association of the *SOX2-OT* gene with bipolar disorders comorbid with eating disorders. Regions containing genes involved in neurodevelopment were also associated with this group. Moreover, Remnelius et al. (22), Carpita et al. (23), Saure et al. (24), Westwood et al. (25) reported a link between ASD and anorexia nervosa, and suggested a specific genetic link between anorexia nervosa and ASD.

**Table 1:** Effects of synthetic estrogens (DES, EE) on psychiatric disorders *in-utero* exposed children (2^nd^ generation).

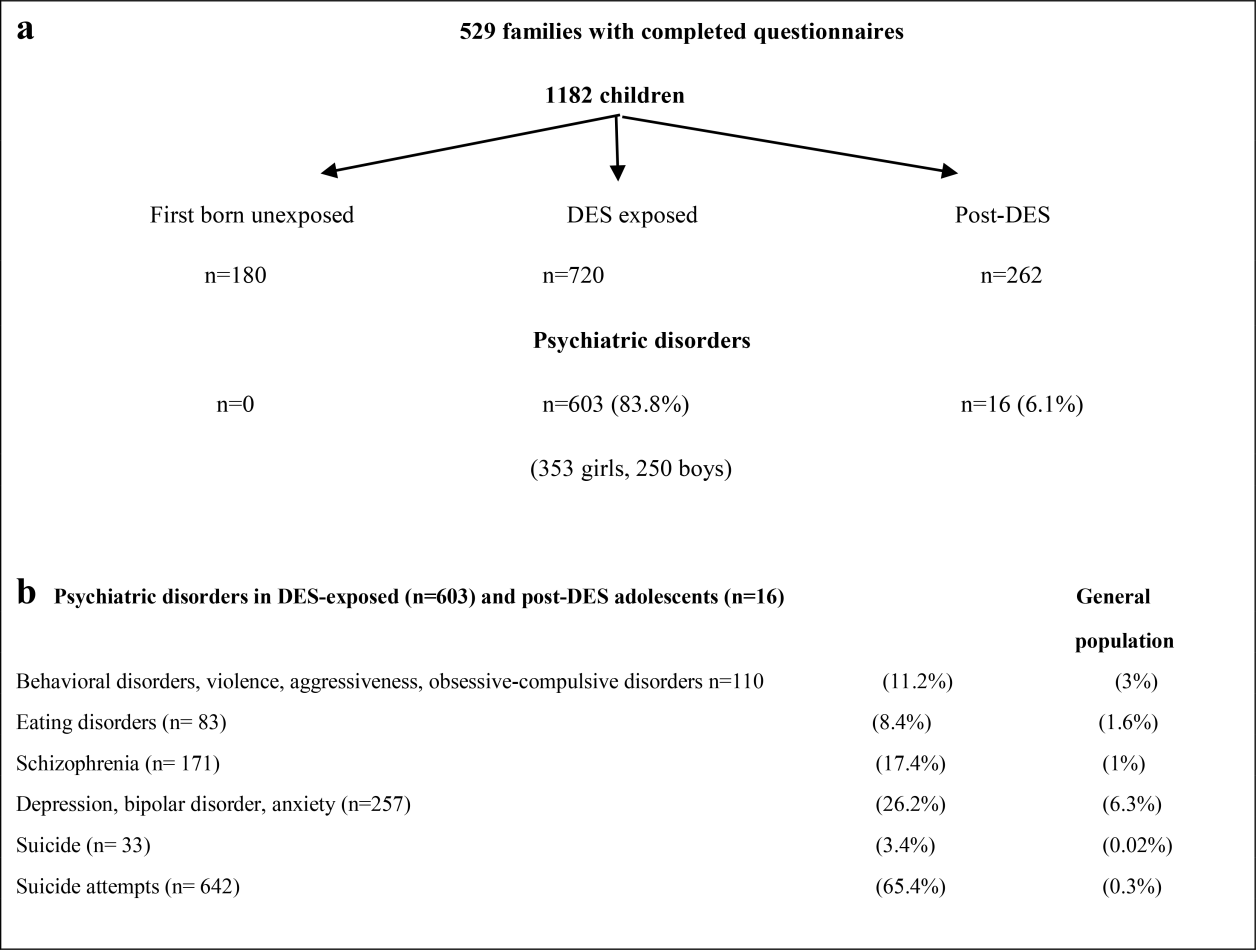			

(a) Total number ofpsychological/psychiatric disorders among the 982 DES/EE-exposed and post-DES unexposed children. The first unexposed children (n= 180) were used as intrafamilial control. With permission by Taylor & Francis Ltd (www.tandfonline.com). (b) Psychiatric disorder rate among the 982DES/EE *in-utero* exposed and post-DES children and in the general population. Note the particularly high number of suicides and suicideattempts. With permission by Taylor & Francis Ltd (www.tandfonline.com).

### Estrogen impact on neurodevelopment: Epigenetic alterations that can be transmitted to the subsequent generations

Data and families from the Hhorages-France cohort were used also to study the molecular basis of the causal link between *in-utero* exposure to synthetic estrogens and the appearance of psychotic disorders, such as schizophrenia and bipolar disorders (26). From this cohort, 69 siblings (n=37 *in-utero* exposed to DES: 12 boys and 25 girls; and n=32 unexposed: 12 boys and 20 girls) from 30 families were selected for DNA methylation analysis in peripheral blood samples. This analysis highlighted specific differences in DNA methylation at the *ZFP57* and *ADAMTS9* genes between exposed and unexposed participants that were correlated with psychotic disorders, particularly schizophrenia, depression and bipolar disorders. In this pioneering work, the authors analyzed the methylation variations of 411,947 CpG sites/per genome in peripheral blood samples, because it was previously reported that the methylation profiles in brain and blood samples are rather similar (27). Controls were unexposed elders (“informative family”) and another cohort of unexposed adolescents who became schizophrenic and were followed at St. Anne Hospital, Paris (28). On the basis of these results, the authors suggested that in exposed individuals, psychosis is associated with specific methylome modifications (i.e. hyper methylation) that modify the gene activity and consequently could influence neurodevelopment and neuroplasticity. One of the genes in which methylation changes have been observed is *ADAMTS9* that encodes a disintegrin and metalloproteinase with thrombospondin motifs 9 (ADAMTS9). Members of the ADAMTS family of proteoglycanases have been implicated in proteoglycan cleavage, organ shape control during development, and angiogenesis inhibition (29, 30). Specifically, sexual organ shape alteration, which is often observed in children *in-utero* exposed to DES, is caused by deregulation of procollagen N-proteinases of the ADAMTS family that regulate collagen fibril assembly and of aggrecanases, other ADAMTS members that regulate the cleavage of extracellular matrix proteoglycans, thus modifying the extracellular matrix structure and function. In addition, the aberrant activation of *ADAMTS9* could induce premature rupture of gestational membranes before the end of gestation (31). Moreover, Tokmak et al. (32) and Wan et al. (33) suggested a link between *ADAMTS9* and endometriosis. Therefore, deregulated expression of *ADAMTS* proteoglycanases might influence fetal neuroplasticity by affecting the extracellular matrix structure and function in nervous system cells (34, 35).

### Estrogens and progestogens may induce autism spectrum disorders

Baron-Cohen et al. (36) analyzed by liquid chromatography and mass spectrometry analysis amniotic fluid samples from 128 boys with ASD from the Danish Historic Birth Cohort. They found (36) that the fetal concentrations of sex hormones (especially progesterone and 17-α-hydroxyprogesterone) were elevated compared with matched control and provided the first evidence of increased fetal steroidogenic activity in autism. Using the same cohort and methods, the authors analyzed the prenatal levels of estrogens and progesterone in the amniotic fluid of boys with (n=98) and without autism (n=177). They observed that the prenatal estrogen and progesterone concentrations were higher in amniotic fluid of boys who developed autism. They concluded that prenatal estrogen alterations influence brain development and sexual differentiation and also contribute to the risk of autism.

Biochemical and molecular studies using limbic tissues from the offspring of progestin-injected pregnant rats (11) showed that prenatal progestin exposure induces autism-like behavior in the offspring through estrogen receptor β (ERβ) suppression in the brain, suggesting that progestin may be implicated in ASD development. Specifically, pregnant rats received LNG alone, LNG plus EE, or EE alone from day 1 to day 21 of gestation. Half of the offspring was used for *in vivo* behavioral studies and the other half for cytochemical, immunological and biochemical studies of the dissected limbic compartment (amygdala, hippocampus and hypothalamus). The authors found that both LNG and LNG/EE significantly increased DNA methylation at the ERβ promoter in amygdala neurons from 10-week-old male and female offspring.

These results in an animal model were then confirmed in a large case-control epidemiological study (**Table 2**) showing the association of prenatal progestin exposure with ASD (12). The authors investigated a large population in Hainan, an island of 8 million people in southern China. Among 37,863 children (0–6 years of age), they selected 235 children with ASD and 682 matched controls. They observed that the following mother-related factors were strongly associated with ASD prevalence: use of progestin to prevent abortion (15.3%), use of progestin contraceptives at conception time (11%), and prenatal consumption of progestin-contaminated seafood during the first trimester of pregnancy (all mothers). The association with the consumption of contaminated seafood by mothers is particularly striking. Indeed, according to the authors, currently in China, combined oral contraceptives are widely used by the seafood industry for pregnancy prevention in fish and shrimps because they can grow faster and become fatter if they do not lay eggs. This may partly explain the increasing incidence of ASD in China (and in other countries). Therefore, pregnant women must be especially careful and must avoid eating oral contraceptive-contaminated seafood. To confirm these findings, the authors gave to pregnant rats synthetic progestin (norethindrone)-treated zebrafish as food, and found that their offspring showed autism-like behavior. The authors concluded that prenatal progestin exposure may be associated with ASD development.

**Table 2:** Progestin exposure and ASD: An epidemiological study in a large Chinese population.

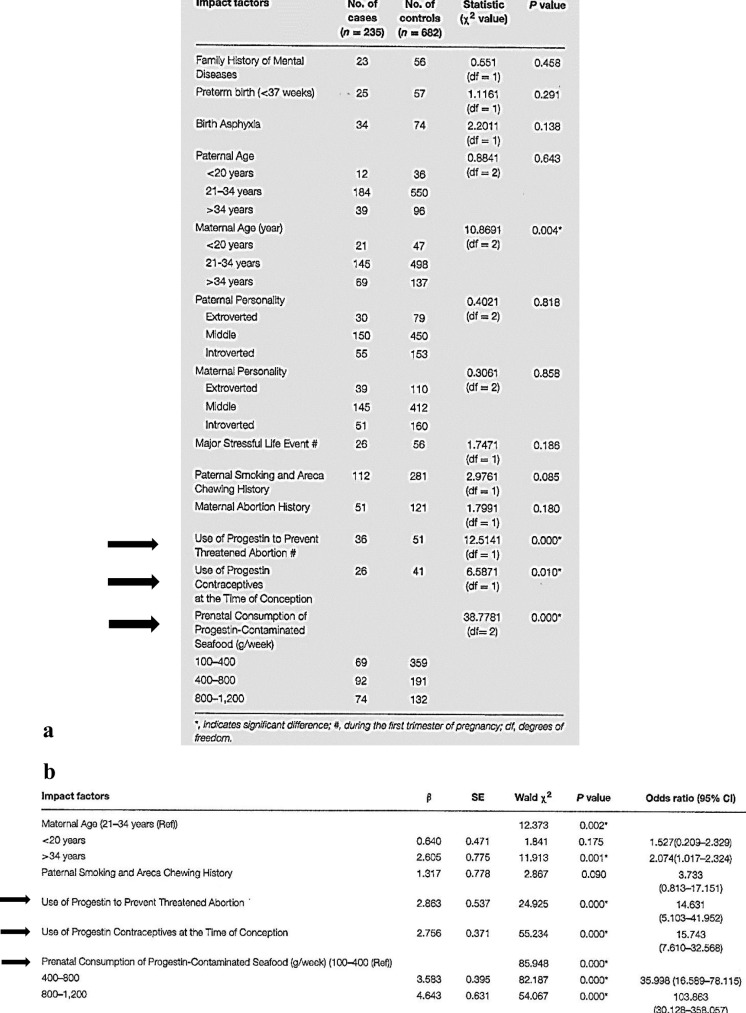						

*, indicates significant differences; #, during the first trimester of pregnancy; df, degrees of freedom

(a) Potential impact factors of progestin exposurefor ASD in a Chinese population-based case-control study. The three major exposure sources during pregnancy are indicated with black arrows.©Li et al. (12). (b) Multivariate regression analysis of the impact factors on ASD prevalence after progestin *in-utero* exposure. Note the high oddsratio for the three major progestin exposure sources (black arrows). ©Li et al. (12). With permission.

### DES, autism and multi- and trans-generational effects

In a large multi-center study on prenatal DES exposure, Titus-Ernstoff et al. (14) observed somatic defects among 4,029 sons (genitourinary anomalies, risk of infertility and testicular cancer) and 3,808 daughters (infertility, reproductive tract anomalies, pregnancy loss, premature delivery, vaginal clear cell adenocarcinoma, risk of breast and cervical cancer) who were *in-utero* exposed. Moreover, for the first time, they reported birth defects in the offspring of prenatally exposed and unexposed children. They also reported the first molecular studies in female mice showing that exposure to DES results in epigenetic alterations.

**Figure 2:**
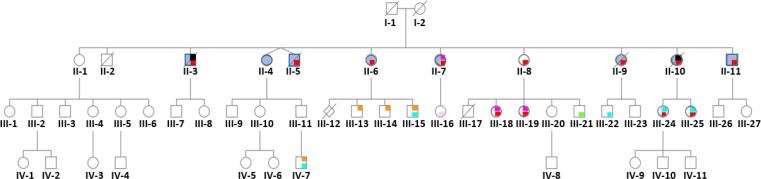
Pedigree of an informative family where the mother (I-2) was treated with DES (30 mg/day) for 3 months after each delivery to inhibit lactation. Only the first child (II-1) was not exposed to DES *in utero*. Daughter II-1 and her descendants do not have any psychiatric disorder. No history of psychiatric disorders was reported for the maternal and paternal sides. red = bipolar disorder, yellow = psychosis borderline, blue = attempted suicide(s), black = suicide, orange = autism spectrum disorder, light blue = dyspraxia and learning disabilities, light pink = mood and sleep disorders, green = behavioral disorders, fuchsia = eating disorders. ^©^Soyer-Gobillard et al. (42) IJERPH, 18, 9965, MDPI.

In 2011, using data on 529 families from the Hhorages-France cohort, Kalfa et al. (37) found that the percentage of children with hypospadias was more than doubled in the grandsons compared with the sons of women who took DES during pregnancy (8.2% vs 3.5%), showing DES multigenerational effect on genital malformations. Rivollier et al. (26) also proposed a possible transgenerational epigenetic effect. These findings confirm the work by Skinner’s group (38) on the epigenetic transmission of the alterations caused by endocrine disruptors and on the epigenetic transgenerational inheritance of diseases. More recently, in a cohort study on DES-linked neurodevelopmental effects that included 47,540 women spanning three generations, Kioumourtzoglou et al. (39) reported attention-deficit/hyperactivity disorder (ADHD) in the third generation of children. ADHD etiology involves a combination of genetic and environmental risk factors. The authors concluded that “DES exposure is associated with multigenerational neurodevelopmental disorders”. Similarly, Gaspari et al. (40, 41) described DES effects on somatic disorders in grandchildren (i.e. primary clear cell carcinoma of the cervix in an 8-year-old granddaughter and endometriosis in granddaughters and possibly one great-granddaughter). Recently, we described in an informative family of 11 children (the elder not exposed was the control), the impact of DES prescribed to suppress lactation for 3 months on the children born after such treatment and their progeny (genealogic tree of this informative family in **Figure 2**), by focusing on psychiatric disorders (bipolar disorders, suicide attempts and suicide, eating disorders) (42, 43). These psychiatric disorders were associated with somatic disorders, such as endometriosis and hypospadias (**Table 3**). Importantly, in the third generation, 10/19 DES-exposed grandchildren had psychiatric disorders, especially ASD, Asperger syndrome or ASD without Asperger syndrome (in boys) and bipolar disorders for girls, associated with dyspraxia and learning disabilities, mood and behavioral disorders and eating disorders, as well as somatic comorbidities. In the fourth generation (n=7 DES-exposed great-grandchildren, aged between 0 and 18 years), one child had dyspraxia and ASD. In the family of the non-exposed daughter, no psychiatric or somatic disorders were reported (**Figure 2** and **Table 3**).

**Table 3:** Psychiatric disorders (columns 2 and 3) and comorbidities (column 4) in the four generations of an informative family (column 1) whose mother (first generation) took DES for 3 months after each delivery (see **Figure 2**).

Patient	Psychiatric disorder	Diagnosis	Associated non-psychiatric disorders	Suicide attempt(s)	Death by suicide
**II-1 ♀**	no	no	no	no	no
**II-2 ♂ **	no	no	Uvula bifida, death due to ruptured congenital brain aneurysm at 26 years of age	no	no
**II-3 ♂ **	yes	Bipolar disorder, chronic alcoholism	Deafness of left ear	yes	yes (at 42 years of age)
**II-4 (♀ twin)**	yes	Eating disorder	Endometriosis	no	no
**II-5 (♂ twin)**	yes	Bipolar disorder, chronic alcoholism	Chronic cirrhosis death at 51 years of age	yes	no
**II-6 ♀**	yes	Bipolar disorder	Endometriosis, inverted kidney/bladder	yes	no
**II-7 ♀**	yes	Bipolar disorder, chronic alcoholism, eating disorder	Endometriosis, sacrococcygial teratoma, rectal adenocarcinoma, breast cancer	yes	no
**II-8 ♀**	yes	Bipolar disorder, chronic alcoholism	Endometriosis, fibromyalgia, obesity	yes	no
**II-9 ♀**	yes	Bipolar disorder, chronic alcoholism	Endometriosis	yes	no
**II-10 ♀**	yes	Bipolar disorder, chronic alcoholism	Endometriosis	yes	yes (at 50 years of age)
**II-I1 ♂**	yes	Bipolar disorder, chronic alcoholism	no	yes	no
**III-1 ♀**	no	no	no	no	no
**III-2 ♂**	no	no	no	no	no
**III-3 ♂**	no	no	no	no	no
**III-4 ♀**	no	no	no	no	no
**III-5 ♀**	no	no	no	no	no
**III-6 ♀**	no	no	no	no	no
**III-7 ♂**	no	no	no	no	no
**III-8 ♀**	no	no	no	no	no
**III-9 ♂**	no	no	no	no	no
**III-10 ♀**	no	no	Endometriosis	no	no
**III-11 ♂**	no	no	Hypospadias	no	no
**III-12 ♂**	/	Very premature baby, anencephaly, deceased	/	/	/
**III-13 ♂**	X	ASD, Asperger syndrome	Hypospadias	no	no
**III-14 ♂**	X	ASD, Asperger syndrome	no	no	no
**III-15 ♂**	X	ASD, learning disorder (dyspraxia)	no	no	no
**III-16 ♀**	X	Mood and sleep disorders	Endometriosis	no	no
**III-17 ♂**	/	Very premature baby, (deceased at day 3 post-partum)	/	/	*/*
**III-18 ♀**	X	Bipolar disorder, eating disorders	Endometriosis	no	no
**III-19 ♀**	X	Bipolar disorder, eating disorders	Endometriosis	no	no
**III-20 ♀**	no	no	Endometriosis	no	no
**III-21 ♂**	X	Behavioral disorders	no	no	no
**III-22 ♂**	X	Learning disorder (dyspraxia)	no	no	no
**III-23 ♂**	no	no	no	no	no
**III-24 ♀**	X	Bipolar disorder, learning disorder	Endometriosis	no	no
**III-25 ♀**	X	Bipolar disorder, learning disorder	Endometriosis	no	no
**III-26 ♂**	no	no	no	no	no
**III-27♀**	no	no	no	no	no
**IV-1 ♀**	no	no	no	no	no
**IV-2 ♂**	/	/	/	/	/
**IV-3 ♀**	no	no	no	no	no
**IV-4 ♂**	/	no	no	no	no
**IV-5 ♀**	no	no	no	no	no
**IV-6 ♀**	no	no	no	no	no
**IV-7 ♂**	yes	ASD, learning disorder (dyspraxia)	no	no	no
**IV-8 ♂**	/	/	/	/	*/*
**IV-9 ♀**	/	/	/	/	*/*
**IV-10 ♂**	/	/	/	/	*/*
**IV-11 ♂**	/	/	/	/	*/*

There are very few studies on the multigenerational (and likely transgenerational) effects of DES in human diseases. It has been hypothesized that ASD originate *in utero* due to molecular perturbations of the developing brain. Ladd-Acosta et al. (44) in a pilot post-mortem study found that *ZFP57* is more methylated in cerebellar tissue samples of women with autism than unrelated controls. In Sprague-Dawley rats, Zou et al. (11) detected DNA hypermethylation on the promoter of the gene encoding ERβ in the amygdala of the offspring *in-utero* exposed to LNG alone or the LNG/EE combination. Interestingly, this effect was less important in female than male rats, as observed also in humans, in agreement with our own recent observations in four generations (42) (**Figure 2**; **Table 3**). In a post-mortem study, Corley et al. (45) showed significant DNA methylation defects in the brain, supporting the hypothesis of an early developmental origin of ASD. In our recent works (42, 46), we confirmed the findings by Kiourmoutzoglou et al. (39) on the multi-generational and very probably trans-generational impact of DES on neurodevelopment. This strengthens the hypothesis that *in-utero* exposure to DES (and to other endocrine disruptors) contributes to the pathogenesis of psychiatric disorders, particularly autistic disorders (47, 48). According to the theory proposed by Nilsson and Skinner (49), epigenetic alterations (epimutations) induced by environmental insults (e.g. exposure to toxicants) and their transgenerational (germline-mediated) inheritance increase disease susceptibility. Indeed, besides the pregnant mother (F0 generation) who is directly exposed to the insult, also the fetus (F1) and its germline are exposed (**Figure 1**) (50). These exposed germ cells will give rise to the F2 generation (grandchildren). Therefore, the first generation without direct environmental exposure is the F3 generation (great-grandchildren). The F3 generation may exhibit transgenerational inheritance of disease susceptibility. Several epimutations can be implicated in this non-genetic inheritance (48): DNA and RNA methylation changes, histone modification alterations, non-coding RNAs and extracellular vesicles. It has been already shown that upon DES exposure, the *ZFP57* and *ADAMTS9* genes become hypermethylated. More studies are needed to identify other epimutations linked to *in-utero* exposure to DES.

### Concomitant neurodevelopmental disorders and genital malformations

Concomitant neurodevelopmental impairment and male genital malformations (disorders of sex development, DSD) have been observed in children exposed *in utero* to DES. Chen et al. (51) reported that boys with cryptorchidism were more likely to receive a diagnosis of ASD (HR 1.24). Similarly, using nation-wide Israeli healthcare data, Rotem et al. (52) highlighted a correlation between male genital malformations and ASD (odds ratio of 1.62) (52). The authors suggested that prenatal androgen defects could be implicated in ASD etiology. We identified several girls and boys with concomitant ASD and DSD in the Hhorages-France cohort (preliminary data) (**Table 3**). Moreover, Bodur et al. (53) recently reported that among 432 boys with ASD, 4.4% had a genital abnormality. Similarly, Pham et al. (54) reviewed data on 447 male children with ASD. They detected genital malformations in 68.5% of them, a much higher rate than previously reported.

## Key findings on DES effects from this review

- Psychiatric effects of estrogens and progestins in *in-utero* exposed children were analyzed using data from a French cohort. Comparisons of groups exposed to different synthetic progestins or estrogens (DES, EE) revealed that the frequency of psychiatric disorders (schizophrenia, bipolarity, eating disorders) is the same whatever the drug.
- A study on the molecular mechanism of estrogen action in the brain of *in-utero* exposed children showed that psychosis was correlated with specific methylome modifications (hypermethylation) in two genes implicated in the neurodevelopment, thus highlighting an epigenetic mechanism.
- Progestin may contribute to ASD development *via* an epigenetic mechanism (hypermethylation of the ERβ gene promoter in the amygdala). A large Chinese epidemiological study showed that many mothers of children with ASD were treated or contaminated by progestins and by oral contraceptive components during pregnancy.
- Analysis of the amniotic fluid in a Danish cohort of boys that included also children with ASD showed that prenatal exposure to estrogens, or other prenatal sex steroids (e.g. progestins) contributes to autism.
- A multigenerational and possibly transgenerational effect of DES on neurodevelopment and psychiatric disorders had been observed, especially for ASD.

## Conclusions

All these results, particularly those reported by Zou et al. (11) and Li et al. (12), support the hypothesis formulated by Kim Strifert (55, 56) on the link between oral contraceptives and higher ASD risk in children (in 2015, 1 in 68 (56) vs in 2018, 1 in 44 children (57) were diagnosed with ASD in the USA. Strifert hypothesized that the estrogen and progestogen used in oral contraceptives act as endocrine disruptors. Specifically, they may affect germ cells/oocytes and give rise to a potent risk factor that helps to explain the recent increase in ASD prevalence. Moreover, such synthetic hormones might cause epigenetic changes. For instance, EE, a known endocrine disruptor, may trigger DNA methylation of the ERβ gene, leading to its downregulation and impaired estrogen signaling in the progeny brain. In 2015, Strifert hypothesized also that the deleterious effects of estrogens and progestogens could be transgenerational.

Therefore, in the absence of large-scale epidemiological studies, the principle of precaution must be imperatively retained for the protection of the future generations: pregnant or not, women should be extremely vigilant about any kind of synthetic hormone therapy. In 2004, the great biologist and epidemiologist T.E. Colborn wrote (15): “The fetus can be protected from endocrine disruptors, whatever they are, only at the zero dose”.

## Author’s note

Association HHORAGES-France, a patient association, is registered at the Epidemiological Portal of French Health Databases INSERM (French National Institute for Medical Research) and AVIESAN (National Alliance for Life Sciences and Health) (epidemiologiefrance.aviesan.fr).

## Author contributions

M-OS-G. drafting the manuscript; LG, PC and CS: revising the manuscript critically for intellectual content. All authors contributed to the article and approved the submitted version.

## Funding

The authors received specific funding for this work from Hhorages-France Association.

## Acknowledgments

This work could not continue without the daily ongoing support of the Hhorages-France families and board especially Mrs. Mauricette P. and Yette B. Authors warmly thank Mr Simon Ball, Independent Autism/ADHD researcher, for his support and encouragements during the writing of the chapter concerning progestogen damages (9). Authors also thank Elisabetta Eandermarcher for corrections and relevant suggestions in the manuscript as well as Professor Paul Yao for invitation, encouragements and permission to reuse some of his team’s results in **Table 2**.

## Conflict of interest

M-OS-G is a researcher, president of the HHORAGES-France Association and a mother concerned with DES and other synthetic hormones.

The remaining authors declare that the research was conducted in the absence of any commercial or financial relationships that could be construed as a potential conflict of interest.

HHORAGES-France Association is financed exclusively by subscriptions and donations.

## Publisher’s note

All claims expressed in this article are solely those of the authors and do not necessarily represent those of their affiliated organizations, or those of the publisher, the editors and the reviewers. Any product that may be evaluated in this article, or claim that may be made by its manufacturer, is not guaranteed or endorsed by the publisher.

## Abbreviations

*ADAM TS9* gene, Implicated in the control of organ shape (uterus and reproductive organs) and CNS development; ADHD, Attention-Deficit/Hyperactivity Disorder; ASD, Autism Syndrome Disorders; DES, Diethylstilbestrol; DSD, Disorders of Sex Development; DMR, Differentially Methylated Region; EE, Ethinylestradiol; ERβ, Estrogen Receptor β; CpG, Cytosine near Guanine LNG, Levonorgestrel (a progestogen); Progestins, Synthetic forms of the body naturally-occurring hormone progesterone.; ZFP57, Gene located on chromosome 6 and expressed very early in development. It is a transcription regulator, directly related to methylation and neurodevelopment.

## References

[1] Tournaire M Epelboin S Devouche E. [Diethylstilbestrol story]. Therapie (2014) 69:101–14. doi: 10.2515/therapie/2014012 24698194

[2] Zamora-Leon P. Are the effects of DES over? A tragic lesson from the past. Int J Environ Res Public Health (2021) 18(19):10309. doi: 10.3390/ijerph181910309 34639609 PMC8507770

[3] Kebir O Krebs MO. Diethylstilbestrol and risk of psychiatric disorders: a critical review and new insights. World J Biol Psychiatry (2012) 13:84–95. doi: 10.3109/15622975.2011.560280 21428730

[4] O'Reilly EJ Mirzaei F Forman MR Ascherio A. Diethylstilbestrol exposure *in utero* and depression in women. Am J Epidemiol (2010) 171:876–82. doi: 10.1093/aje/kwq023 PMC2877444 20332145

[5] Soyer-Gobillard MO Paris F Gaspari L Courtet P Sultan C. Association between fetal DES-exposure and psychiatric disorders in adolescence/adulthood: Evidence from a French cohort of 1002 prenatally exposed children. Gynecol Endocrinol (2016) 32:25–9. doi: 10.3109/09513590.2015.1063604 26172930

[6] Soyer-Gobillard MO Sultan C. Behavioral and somatic disorders in children exposed *in utero* to synthetic hormones: A testimony-case study in a French family troop. In: M. S, editor. State of the art of therapeutic endocrinology. Japan: InTech (2012). p. 67–86.

[7] Soyer-Gobillard MO Gaspari L Courtet P Puillandre M Paris F Sultan C. Neurodevelopmental disorders in children exposed *in utero* to synthetic progestins: Analysis from the national cohort of the Hhorages association. Gynecol Endocrinol (2019) 35:247–50. doi: 10.1080/09513590.2018.1512968 30626235

[8] Yenkoyan K Grigoryan A Fereshetyan K Yepremyan D. Advances in understanding the pathophysiology of autism spectrum disorders. Behav Brain Res (2017) 331:92–101. doi: 10.1016/j.bbr.2017.04.038 28499914

[9] de la Torre-Ubieta L Won H Stein JL Geschwind DH. Advancing the understanding of autism disease mechanisms through genetics. Nat Med (2016) 22:345–61. doi: 10.1038/nm.4071 PMC5072455 27050589

[10] Havdahl A Niarchou M Starnawska A Uddin M van der Merwe C Warrier V. Genetic contributions to autism spectrum disorder. Psychol Med (2021) 51:2260–73. doi: 10.1017/S0033291721000192 PMC8477228 33634770

[11] Zou Y Lu Q Zheng D Chu Z Liu Z Chen H. Prenatal levonorgestrel exposure induces autism-like behavior in offspring through ERbeta suppression in the amygdala. Mol Autism (2017) 8:46. doi: 10.1186/s13229-017-0159-3 28824796 PMC5561609

[12] Li L Li M Lu J Ge X Xie W Wang Z. Prenatal progestin exposure is associated with autism spectrum disorders. Front Psychiatry (2018) 9:611. doi: 10.3389/fpsyt.2018.00611 30510526 PMC6252360

[13] Perrotti L. I.. Estrogen and Progesterone Receptor Signaling and Action. In: Gene Regulation, Epigenetics and Hormone Signaling, Mandal S. S. (Ed.). Hoboken, New Jersey, USA: Wiley-UCH Verlag GmbH & Co. KGaA. (2017). doi: 10.1002/9783527697274.ch11

[14] Titus-Ernstoff L Troisi R Hatch EE Palmer JR Hyer M Kaufman R. Birth defects in the sons and daughters of women who were exposed *in utero* to diethylstilbestrol (DES). Int J Androl (2010) 33:377–84. doi: 10.1111/j.1365-2605.2009.01010.x PMC2874639 20002218

[15] Colborn T. Neurodevelopment and endocrine disruption. Environ Health Perspect (2004) 112:944–9. doi: 10.1289/ehp.6601 PMC1247186 15198913

[16] Soyer-Gobillard MO Gaspari L Sultan C. Evidence for a link between *in utero* exposure to synthetic estrogens and progestins and mental disorders: A long and crucial history. In: A.L. Woolfolk R Durbano F Irtelli F, editors. Psychopathology : An international and interdisciplinary perspective. London: IntechOpen (2020). p. 7–22.

[17] Soyer-Gobillard MO Gaspari L Yao P Sultan Ch. Prenatal exposure to progestins: Impact on neurodevelopment of the child. In: P.V. Martin C Rajendram R, editors. Factors affecting neurodevelopment. London: Academic Press (2021). p. 395–408.

[18] Baker JH Schaumberg K Munn-Chernoff MA. Genetics of anorexia nervosa. Curr Psychiatry Rep (2017) 19:84. doi: 10.1007/s11920-017-0842-2 28940168 PMC6139670

[19] Godart N Radon L Curt F Duclos J Perdereau F Lang F. Mood disorders in eating disorder patients: Prevalence and chronology of ONSET. J Affect Disord (2015) 185:115–22. doi: 10.1016/j.jad.2015.06.039 26162282

[20] Seeman MV. Eating disorders and psychosis: Seven hypotheses. World J Psychiatry (2014) 4:112–9. doi: 10.5498/wjp.v4.i4.112 PMC4274583 25540726

[21] Liu X Bipolar Genome S Kelsoe JR Greenwood TA. A genome-wide association study of bipolar disorder with comorbid eating disorder replicates the SOX2-OT region. J Affect Disord (2016) 189:141–9. doi: 10.1016/j.jad.2015.09.029 PMC4640946 26433762

[22] Lundin Remnelius K Neufeld J Isaksson J Bolte S. Eating problems in autistic females and males: A Co-twin control study. J Autism Dev Disord (2022) 52:3153–68. doi: 10.1007/s10803-021-05198-z PMC9213283 34292489

[23] Carpita B Muti D Cremone IM Fagiolini A Dell'Osso L. Eating disorders and autism spectrum: Links and risks. CNS Spectr (2022) 27:272–80. doi: 10.1017/S1092852920002011 33161925

[24] Saure E Laasonen M Raevuori A. Anorexia nervosa and comorbid autism spectrum disorders. Curr Opin Psychiatry (2021) 34:569–75. doi: 10.1097/YCO.0000000000000742 34419968

[25] Westwood H Eisler I Mandy W Leppanen J Treasure J Tchanturia K. Using the autism-spectrum quotient to measure autistic traits in anorexia nervosa: A systematic review and meta-analysis. J Autism Dev Disord (2016) 46:964–77. doi: 10.1007/s10803-015-2641-0 PMC4746216 26542816

[26] Rivollier F Chaumette B Bendjemaa N Chayet M Millet B Jaafari N. Methylomic changes in individuals with psychosis, prenatally exposed to endocrine disrupting compounds: Lessons from diethylstilbestrol. PloS One (2017) 12:e0174783. doi: 10.1371/journal.pone.0174783 28406917 PMC5390994

[27] Kaminsky Z Tochigi M Jia P Pal M Mill J Kwan A. A multi-tissue analysis identifies HLA complex group 9 gene methylation differences in bipolar disorder. Mol Psychiatry (2012) 17:728–40. doi: 10.1038/mp.2011.64 21647149

[28] Kebir O Chaumette B Rivollier F Miozzo F Lemieux Perreault LP Barhdadi A. Methylomic changes during conversion to psychosis. Mol Psychiatry (2017) 22:512–8. doi: 10.1038/mp.2016.53 PMC5378806 27113994

[29] Kelwick R Desanlis I Wheeler GN Edwards DR. The ADAMTS (A disintegrin and metalloproteinase with thrombospondin motifs) family. Genome Biol (2015) 16:113. doi: 10.1186/s13059-015-0676-3 26025392 PMC4448532

[30] Mittaz L Russell DL Wilson T Brasted M Tkalcevic J Salamonsen LA. Adamts-1 is essential for the development and function of the urogenital system. Biol Reprod (2004) 70:1096–105. doi: 10.1095/biolreprod.103.023911 14668204

[31] Li W Zhao X Li S Chen X Cui H Chang Y. Upregulation of TNF-alpha and IL-6 induces preterm premature rupture of membranes by activation of ADAMTS-9 in embryonic membrane cells. Life Sci (2020) 260:118237. doi: 10.1016/j.lfs.2020.118237 32781068

[32] Tokmak A Ozaksit G Sarikaya E Kuru-Pekcan M Kosem A. Decreased ADAMTS-1, -9 and -20 levels in women with endometrial polyps: A possible link between extracellular matrix proteases and endometrial pathologies(). J Obstet Gynaecol (2019) 39:845–50. doi: 10.1080/01443615.2019.1584890 31010360

[33] Wan Y Gu C Kong J Sui J Zuo L Song Y. Long noncoding RNA ADAMTS9-AS1 represses ferroptosis of endometrial stromal cells by regulating the miR-6516-5p/GPX4 axis in endometriosis. Sci Rep (2022) 12:2618. doi: 10.1038/s41598-022-04963-z 35173188 PMC8850595

[34] Lemarchant S Pruvost M Montaner J Emery E Vivien D Kanninen K. ADAMTS proteoglycanases in the physiological and pathological central nervous system. J Neuroinflamm (2013) 10:133. doi: 10.1186/1742-2094-10-133 PMC4228433 24176075

[35] Chelyshev YA Kabdesh IM Mukhamedshina YO. Extracellular matrix in neural plasticity and regeneration. Cell Mol Neurobiol (2022) 42:647–64. doi: 10.1007/s10571-020-00986-0 PMC11441266 33128689

[36] Baron-Cohen S Auyeung B Norgaard-Pedersen B Hougaard DM Abdallah MW Melgaard L. Elevated fetal steroidogenic activity in autism. Mol Psychiatry (2015) 20:369–76. doi: 10.1038/mp.2014.48 PMC4184868 24888361

[37] Kalfa N Paris F Soyer-Gobillard MO Daures JP Sultan C. Prevalence of hypospadias in grandsons of women exposed to diethylstilbestrol during pregnancy: A multigenerational national cohort study. Fertil Steril (2011) 95:2574–7. doi: 10.1016/j.fertnstert.2011.02.047 21458804

[38] Skinner MK. Endocrine disruptor induction of epigenetic transgenerational inheritance of disease. Mol Cell Endocrinol (2014) 398:4–12. doi: 10.1016/j.mce.2014.07.019 25088466 PMC4262585

[39] Kioumourtzoglou MA Coull BA O'Reilly EJ Ascherio A Weisskopf MG. Association of exposure to diethylstilbestrol during pregnancy with multigenerational neurodevelopmental deficits. JAMA Pediatr (2018) 172:670–7. doi: 10.1001/jamapediatrics.2018.0727 PMC6137513 29799929

[40] Gaspari L Soyer-Gobillard MO Paris F Kalfa N Hamamah S Sultan C. Multigenerational endometriosis: Consequence of fetal exposure to diethylstilbestrol? Environ Health (2021) 20:96. doi: 10.1186/s12940-021-00780-5 34452632 PMC8401160

[41] Gaspari L Paris F Cassel-Knipping N Villeret J Verschuur A Soyer-Gobillard MO. Diethylstilbestrol exposure during pregnancy with primary clear cell carcinoma of the cervix in an 8-year-old granddaughter: A multigenerational effect of endocrine disruptors? Hum Reprod (2021) 36:82–6. doi: 10.1093/humrep/deaa267 33147330

[42] Soyer-Gobillard MO Gaspari L Paris F Kalfa N Hamamah S Courtet P. Prenatal exposure to diethylstilbestrol and multigenerational psychiatric disorders: An informative family. Int J Environ Res Public Health (2021) 18(19):9965. doi: 10.3390/ijerph18199965 34639263 PMC8507930

[43] Soyer-Gobillard MO Gaspari L Sultan C. Consequences of *In utero* exposure to synthetic estrogens and progestogens for children and grandchildren. IJCIMR (2021) 14:001–5. doi: 10.55920/IJCIMR.2021.01.0001001

[44] Ladd-Acosta C Hansen KD Briem E Fallin MD Kaufmann WE Feinberg AP. Common DNA methylation alterations in multiple brain regions in autism. Mol Psychiatry (2014) 19:862–71.doi: 10.1038/mp.2013.114 PMC4184909 23999529

[45] Corley MJ Vargas-Maya N Pang APS Lum-Jones A Li D Khadka V. Epigenetic delay in the neurodevelopmental trajectory of DNA methylation states in autism spectrum disorders. Front Genet (2019) 10:907.doi: 10.3389/fgene.2019.00907 31681403 PMC6797928

[46] Soyer-Gobillard MO Gaspari L Sultan C. *In utero* exposure to synthetic hormones: consequences upon exposed children, especially multigenerational transmission. Int J Clin Stud Med Case Rep (2021) 1(1):10004. doi: 10.55920/IJCIMR.2021.01.0001001

[47] Ravel C Kah O. [Endocrine disrupters: Towards an unsatisfying regulation]. Presse Med (2018) 47:943–9. doi: 10.1016/j.lpm.2018.08.001 30217365

[48] Montjean D Neyroud AS Yefimova MG Benkhalifa M Cabry R Ravel C. Impact of endocrine disruptors upon non-genetic inheritance. Int J Mol Sci (2022) 23(6):3350. doi: 10.3390/ijms23063350 35328771 PMC8950994

[49] Nilsson EE Skinner MK. Environmentally induced epigenetic transgenerational inheritance of reproductive disease. Biol Reprod (2015) 93:145. doi: 10.1093/eep/dvy016 26510870 PMC6058737

[50] Shahidehnia M. Epigenetic effects of endocrine disrupting chemicals. J Environ Anal Toxicol (2016) 6:381. doi: 10.4172/2161-0525.1000381

[51] Chen J Sorensen HT Miao M Liang H Ehrenstein V Wang Z. Cryptorchidism and increased risk of neurodevelopmental disorders. J Psychiatr Res (2018) 96:153–61. doi: 10.1016/j.jpsychires.2017.10.006 29065375

[52] Rotem RS Chodick G Davidovitch M Hauser R Coull BA Weisskopf MG. Congenital abnormalities of the Male reproductive system and risk of autism spectrum disorders. Am J Epidemiol (2018) 187:656–63. doi: 10.1093/aje/kwx367 PMC5888926 29452340

[53] Gul H Bodur Ş. Çetinkaya M Taşkıran C Işıldar Y. Findings of Male genital anomalies in a Turkish population with autism spectrum disorders. Anatolian Clinic J Med Sci (2019) 24:72–7. doi: 10.21673/anadoluklin.460738

[54] Pham T Patel A Muquith M Zimmern V Goodspeed K. Abnormal genetic testing in males with concomitant neurodevelopmental disabilities and genital malformation. Pediatr Neurol (2022) 134:72–7. doi: 10.1016/j.pediatrneurol.2022.06.019 35841714

[55] Strifert K. The link between oral contraceptive use and prevalence in autism spectrum disorder. Med Hypotheses (2014) 83:718–25. doi: 10.1016/j.mehy.2014.09.026 25459142

[56] Strifert K. An epigenetic basis for autism spectrum disorder risk and oral contraceptive use. Med Hypotheses (2015) 85:1006–11. doi: 10.1016/j.mehy.2015.09.001 26364046

[57] Maenner MJ Shaw KA Bakian AV Bilder DA Durkin MS Esler A. Prevalence and characteristics of autism spectrum disorder among children aged 8 years - autism and developmental disabilities monitoring network, 11 sites, United States, 2018. MMWR Surveill Summ (2021) 70:1–16. doi: 10.15585/mmwr.ss7011a1 PMC8639024 34855725

